# Fluorescence imaging of in vivo miR-124a-induced neurogenesis of neuronal progenitor cells using neuron-specific reporters

**DOI:** 10.1186/s13550-016-0190-y

**Published:** 2016-04-26

**Authors:** Jaeho Jang, Song Lee, Hyun Jeong Oh, Yoori Choi, Jae Hyouk Choi, Do Won Hwang, Dong Soo Lee

**Affiliations:** Department of Nuclear Medicine, Seoul National University College of Medicine, Seoul, South Korea; Department of Molecular Medicine and Biopharmaceutical Sciences, Graduate School of Convergence Science and Technology, and College of Medicine or College of Pharmacy, Seoul National University, Seoul, South Korea

**Keywords:** miR-124a, Activation of neuronal differentiation, Neuron-specific reporter gene, In vivo fluorescence imaging

## Abstract

**Background:**

Facilitation of the differentiation of the stem cells toward neuronal lineage is crucial for enhancing the differentiation efficacy of grafted stem cells for the possible treatment of neurodegenerative disorders. MicroRNA124a (miR-124a) has been considered as a neuronal lineage regulator, possessing the capability to activate neuronal differentiation. In this study, using a neuronal promoter-based reporter and live-cell fluorescence imaging, we visualized in vitro and in vivo the enhanced neuronal differentiation of neuronal progenitor cells with miR-124a overproduction.

**Methods:**

The neuron specific alpha1 tubulin promoter-driven RFP reporter (pTa1-RFP) was used to trace the miR-124a-induced neuronal differentiation in live cell condition. MiR-124a or miR-scramble in 10 % glucose buffer was mixed with in vivo-jetPEITM and in vivo fluorescence images were obtained daily using Maestro spectral fluorescent imager.

**Results:**

Neurite outgrowth was clearly seen in F11 cells after miR-124a transfection, and immunofluorescence staining showed increase of Tuj1 and NF at 48 hours. When pTa1-RFP-transfected F11 cells were implanted simultaneously with miR-124a into the nude mice, gradually increasing reporter signals and morphological changes indicated neuronal differentiation for 48 hours in live cells in vitro. The miR-124a-treated F11 cells showed higher reporter signals on in vivo fluorescence imaging than miR-scramble-treated cells, which were verified by ex vivo confirmation of Tuj1 and NF expression.

**Conclusions:**

These results indicated that neuronal reporter-based neurogenesis imaging can be used for monitoring miR-124a acting as neuronal activator when miRNA was injected in in vivo PEI-coated form for miRNA-mediated regenerative therapy.

## Background

For the intractable brain diseases, the feasibility of immunotherapy and cell-based therapy have been studied using primates or small animals [1–3]. Therapeutic strategy using stem or progenitor cells was proposed as a possibility to treat neurodegenerative disorders replacing the injured brain tissues with cell implants. Embryonic stem cells, mesenchymal stem cells, and neural stem or progenitor cells were proposed as cell candidates [4–7]. Among these cells, neural stem or progenitor cells were found promising as they are able to differentiate to neurons if implanted [8–11]. However, though these stem cells were highly expected to differentiate into neuronal cells for the success of stem cell treatment, in reality, they rarely differentiate into the desired cell lineage when implanted. Thus, investigators used a variety of potential factors to enhance the differentiation in vivo, such as neurogenin 1 (ngn1), neuroD2 (pro-neural basic helix-loop-helix transcription factors), or microRNAs [12–15].

MicroRNAs participate in post-transcriptional regulation of cellular differentiation by binding to the complementary sequences of target messenger RNAs (mRNAs) or non-coding RNAs and down-regulate their target gene expression. During developmental process, let-7 microRNAs regulate developmental timing or certain microRNAs are over-expressed or under-expressed in diseases such as cancer, metabolic diseases, and cardiovascular disorders [16–20]. MicroRNAs might be used as a tool to control the biological process of interest even for the treatment of neurodegenerative diseases. Among the variety of microRNAs expressed exclusively in the nervous system, miR-124a is abundantly expressed as neuron-specific miRNAs [21–25]. miR-124a was reported to promote neuronal differentiation by triggering brain-specific alternative pre-mRNA splicing [26–30] and was known to participate in neuronal fate determination during spinal cord development [31], and miR-124a was necessary for a proper brain development and axogenesis of dentate granule neurons [32]. miR-124a might be able to promote neuronal differentiation of stem or progenitor cells when co-administered with the stem/progenitor cell implants as a differentiation activator. If proven for its efficacy, injection of miR-124a might enhance the feasibility of using progenitor cell implants in intractable neurological disorders.

Reporter genes have been used as molecular-genetic imaging agents for monitoring various cellular biologic processes [33–35]. For visualization of neuronal differentiation using molecular imaging techniques, neuron-specific promoter-driven optical reporters can be used as simple imaging system to monitor the transition of implanted stem cells into neuronal lineage in vivo. Tα1, a member of tubulin, is expressed abundantly during neuronal development, and Tα1 mRNA is enriched in neurons undergoing active neurite extension [36, 37]. In this study, neuron-specific pTα1 promoter-driven RFP reporter system was adopted to trace the activation of neuronal differentiation in vitro and in vivo induced by the administration of exogenous miR-124a in the implants of neural progenitor or stem cells.

## Methods

### Cell cultures

F11 (hybrid cell line of rat dorsal root ganglion cell with mouse neuroblastoma) and HB1.F3 (an immortalized, clonal human neural stem cell line) were cultured in Dulbecco’s modified Eagle’s medium (DMEM, Invitrogen, Grand Island, NY) supplemented with 10 % fetal bovine serum (FBS, Invitrogen, Grand Island, NY) and 1 % antibiotics (Invitrogen, Grand Island, NY) at 5 % CO_2_ atmosphere.

### MiRNAs and fluorescence reporter gene transfection

To promote neuronal differentiation by miR-124a, 1 × 10^5^ of F11 or F3 cells were seeded in 12-well plates. After 24-h incubation, 50 nM of chemically modified miR-124a or miR-scramble oligomer (Life technologies, Ambion, CA) was transfected into F11 or F3 cells using a Lipofectamine reagent (Invitrogen, Grand Island, NY). For a live cell imaging, 1 × 10^4^ of F11 or F3 cells were seeded in eight-well chamber slide (Nunc, NY). 0.2 μg of pTα1 (Talpha1 promoter)-RFP (kindly donated by Keejung Yoon, Sungyunkwan University) [38] and miRNA oligomer were co-transfected into F11 cells using Lipofectamine.

### Immunofluorescence imaging and neurite outgrowth measurement

Cells were fixed with 4 % paraformaldehyde (USB®, OH) or 20 min at room temperature and incubated in 0.5 % hydrogen peroxide in methanol for 20 min. After phosphate buffered saline (PBS) washing, cells were incubated in 0.1 % Triton X-100 (Samchum Chemical, Seoul). Thirty minutes after blocking with 1:30 dilution of normal goat serum, 1:300 dilution of rabbit neuron-specific class III β-tubulin (Tuj1; Sigma, MO), 1:500 dilution of rabbit neurofilament (NF; Sigma, MO), 1:300 dilution of mouse glial fibrillary acidic protein (GFAP; Cell Signaling, MA), 1:300 dilution of rabbit enolase-1 (Cell Signaling, MA), and 1:300 dilution of mouse BrdU (Cell signaling, MA), these samples were incubated for 24 h at 4 °C. After washing, the cells were incubated for 1 h with 1:400 dilution of Alexa Fluor 488 anti-mouse and Alexa Fluor 555 anti-rabbit secondary antibodies (Invitrogen, CA). Sample slides were prepared with 4′,6-diamidino-2-phenylindole hydrochloride (DAPI) mounting solution (Vector Laboratories, CA). Fluorescence images were obtained, and neurite outgrowths were measured by a Zeiss 510 Meta Laser scanning microscope (LSM; Carl Zeiss Microimaging, NY).

### Fluorescence live cell imaging of F11 transfected with miR-124a

For visualizing the activation of neuronal differentiation induced by the overproduction of miR-124a, 1 × 10^4^ of F11 cells were seeded in each well of eight-well chamber slide and co-transfected with miR-124a (40 nM) and pTα1-RFP (0.2 μg). During 48 h, phase contrast and fluorescence images were obtained in a time-lapse imaging system (Olympus IX81 ZDC microscope, PA). Each image was obtained simultaneously with every 30-min intervals.

### In vivo fluorescence imaging of neuronal differentiation of F11 cells co-administered with miR-124a

**Fig. 1:**
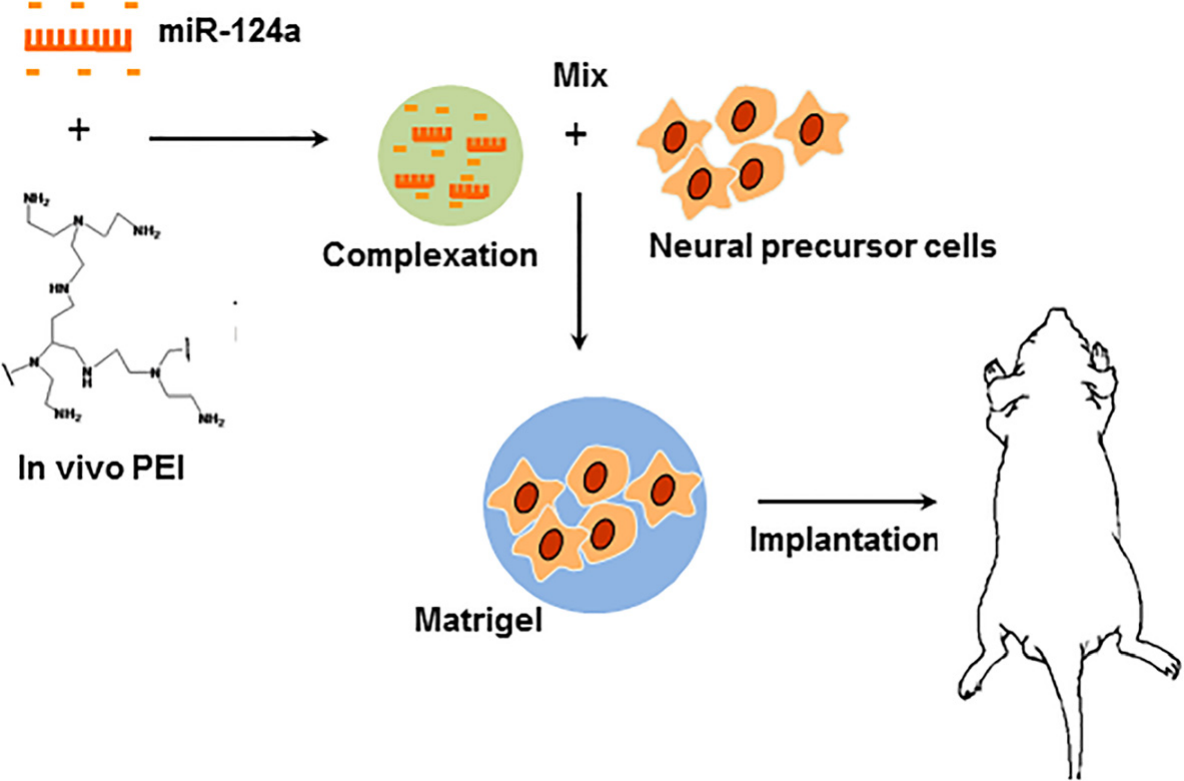
Schematic display of experimental procedure for miR-124a-induced neurogenesis in vivo. Complexation between negatively charged miR-124a oligomer and positively charged in vivo PEI polymer was formed at room temperature, and neural progenitor cells were mixed with this pre-formed complex polymer before cell implantation. Temperature-sensitive matrigel was used to trap the cells within gel scaffold, and this cell/matrigel complex was implanted into the thighs of nude mice

Seven-week-old male BALB/c nude mice (*n* = 3) were used for the in vivo fluorescence imaging and housed under pathogen-free animal environment. Mice were handled according to the ethical and biosafety guidelines issued by the Institutional Animal Care and Use Committee (IACUC: 2014-0066) of Seoul National University Hospital (SNUH). This research was reviewed and finally approved by SNUH committee with qualified animal experience and expertise. Firstly, 9 × 10^6^ of pTα1-RFP-transfected F11 cell pellets were prepared. For the miRNA transfection, 50 nM of miR-124a or miR-scramble in 10 % glucose buffer was mixed with in vivo jetPEI™ (Polyplus-transfection Inc., NY) and incubated for 15 min at RT. The miRNA-polyethylenimine (PEI) complexes were mixed with the prepared F11 cell pellets, and the cells mixed with 150 μl of matrigel (BD Bioscience, CA) were subcutaneously injected into both thighs of nude mice (Fig. 1). After the injections, in vivo fluorescence imaging was daily acquired using Maestro spectral fluorescent imager (Maestro; Cri Inc., MA). At 4 days, the mice skin was removed and fluorescence signals from the region of interest (ROI) were quantitatively analyzed.

### Immunohistochemistry of implanted mass

Implanted mass over the thighs of the nude mice were excised, and these samples containing F11 cells with miR-124a or miR-scramble were fixed with 4 % paraformaldehyde for 20 min at room temperature. The samples were embedded in paraffin block, and the paraffin block was sectioned by a rotary microtome Leica RM2145 (Leica Microsystems, Wetzlar). Ten-micrometer-thick sectioned samples were stained with primary antibodies against 1:100 dilution of rabbit RFP (Abcam Inc., MA), 1:300 dilution of rabbit Tuj1 (Sigma, MO), and 1:500 dilution of rabbit NF (Sigma, MO) overnight at 4 °C. Then, the samples were incubated with 1:400 dilution of Alexa Fluor 488 anti-mouse and Alexa Fluor 488 anti-rabbit secondary antibodies for 1 h. The sample slides were prepared with 4′,6-diamidino-2-phenylindole hydrochloride (DAPI) mounting solution and fluorescence images were obtained by a Zeiss 510 Meta Laser scanning microscope (LSM; Carl Zeiss Microimaging, NY).

## Results

### Morphological changes of F11 cells treated with miR-124a

**Fig. 2:**
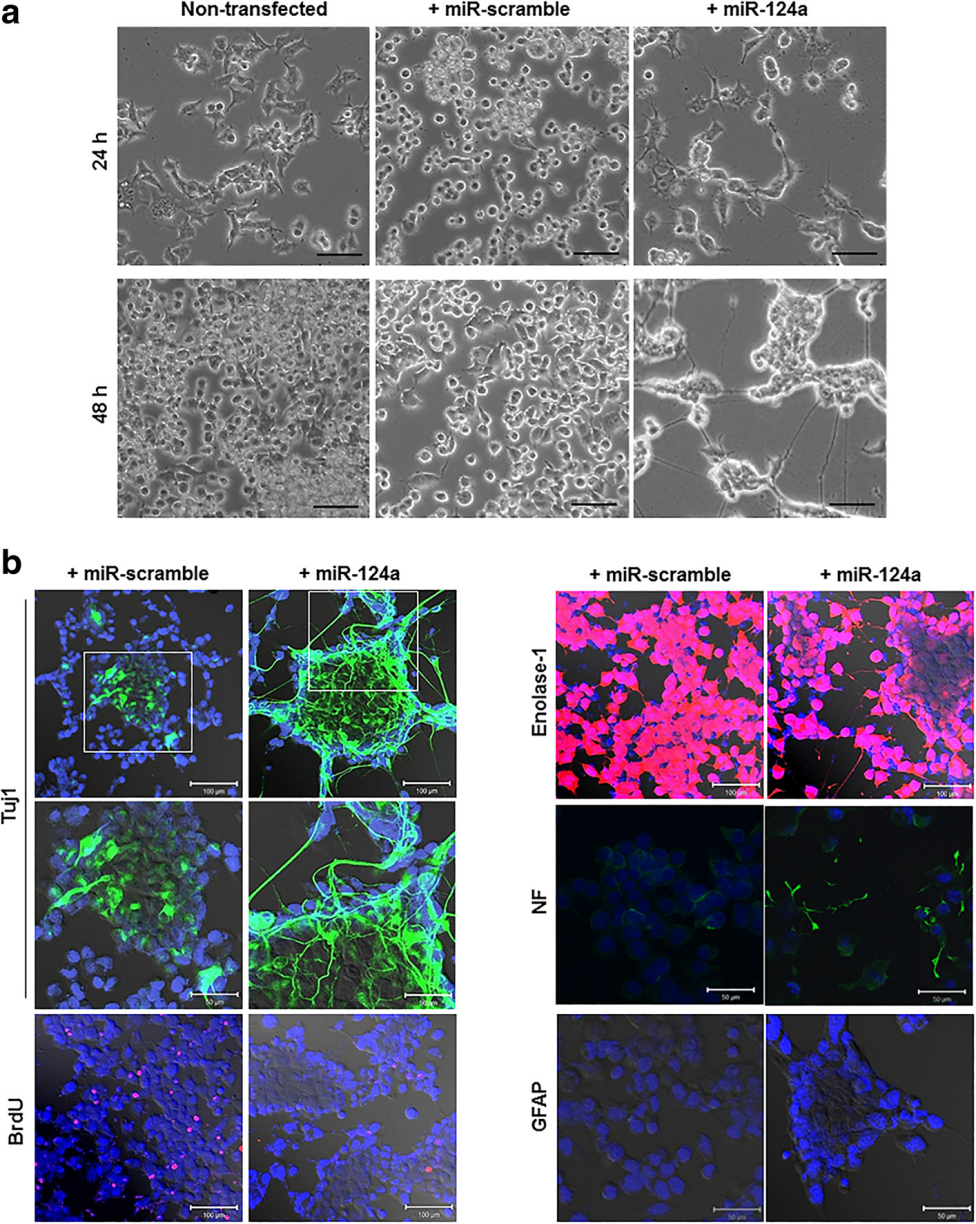
Enhanced neuronal gene expression in F11 cells treated with miR-124a. **a** F11 cells were transfected with miR-124a or miR-scramble oligomer, respectively. Morphological changes were observed at 24 and 48 h after transfection. MiR-124a-transfected cells showed significant neurite outgrowth, compared to non-transfected or miR-scramble-transfected cells. *Scale bars*, 100 μm. **b** F11 cells transfected with either miR-124a or miR-scramble were fixed using 4 % paraformaldehyde for 20 min. Antibodies against enolase, Tuj1, NF, and GFAP were used to evaluate the neuronal differentiation stage. Tuj1 and NF expression increased significantly in miR-124a-transfected F11 cells

To confirm the changes of F11 cells by miR-124a in their morphology toward neuronal characteristics, miR-124a or miR-scramble was transfected into F11 cells at 24 h after cell seeding. At 24 and 48 h post-transfection, significant neurite outgrowth was observed in the miR-124a-transfected group (Fig. 2a). In contrast, miR-scramble-treated and non-treated group showed no morphological changes until 2 days.

### Enhanced expression of neuronal markers induced by miR-124a in F11 and F3 cells

To verify the enhanced expression of neuronal markers of F11 cells induced by the overproduction of miR-124a, we performed immunofluorescence at 48 h after miR-124a transfection using neuronal-specific antibodies Tuj1, NF, enolase, and GFAP. Neuron-specific class III β-tubulin (Tuj1) is present in newly generated immature post-mitotic neurons and differentiated neurons and in some mitotically active neuronal precursors. Neurofilaments (NFs) are the intermediate filaments found specifically in neurons. Tuj1 and NF expressions were significantly higher in miR-124a-treated F11 cells, compared to miR-scramble-treated cells (Fig. 2b). BrdU staining results showed that the population of proliferating cells decreased in miR-124a-treated F11 cells suggesting that miR-124a-induced neuronal differentiation is associated with the decreased proliferation. Enolase-1 is ubiquitously expressed in adult tissues including the brain and liver and elevated in neuroblastoma. As a marker of neuroblastoma, ubiquitously expressing enolase-1 expression was intense in both scramble- and miR-124a-treated F11 cells and glial cell marker GFAP expression was not found in both groups.

**Fig. 3:**
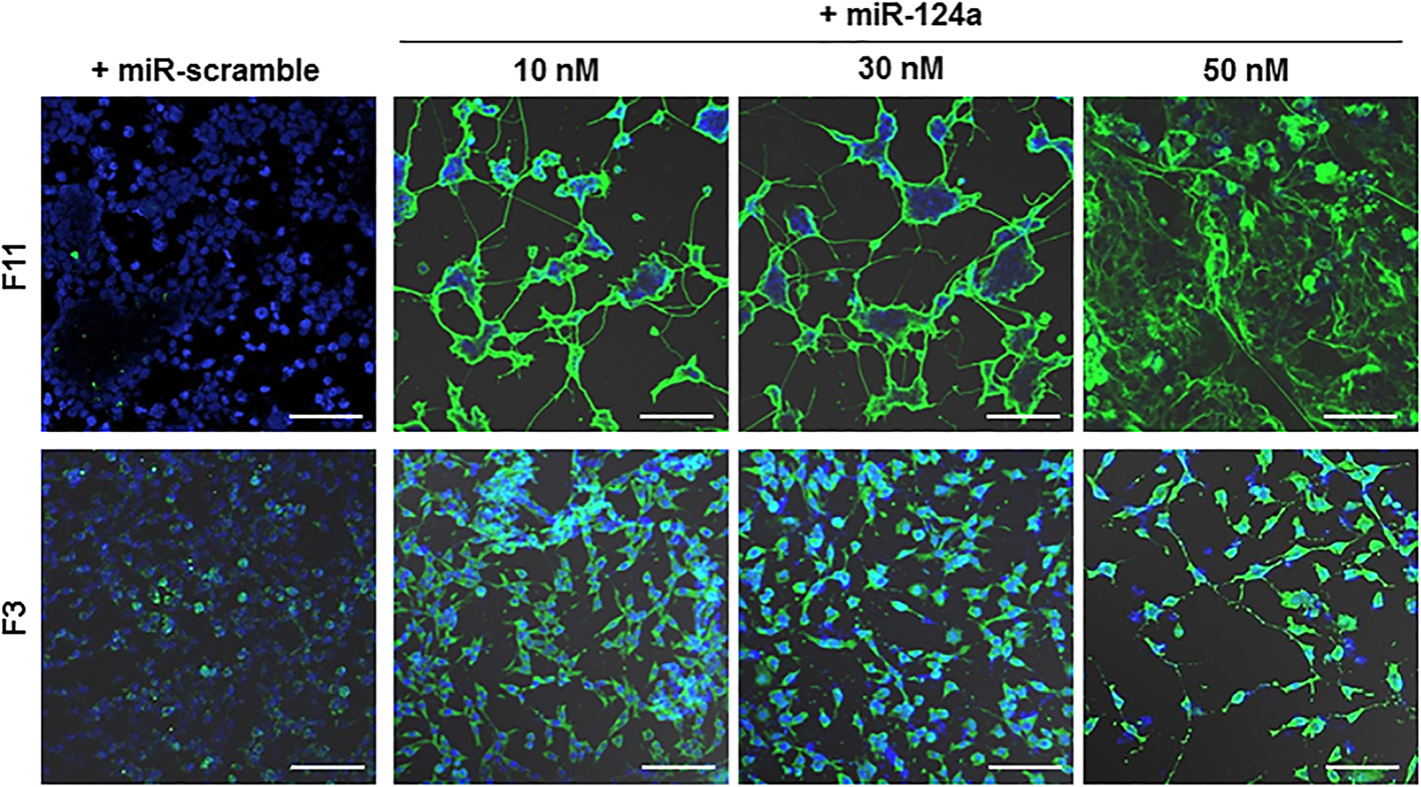
Activation of neuronal differentiation by miR-124a in F11 and F3 cells. Two days after transfection of different dose of miR-124a/miR-scramble (10, 30, and 50 nM), immunofluorescence was done using Tuj1 antibody. Tuj1 expression in both F11 and F3 cells was increased from 10 nM concentration of miR-124a oligomer. Treatment of miR-scramble did not induce Tuj1 expression in both F11 and F3 cells

To examine that F3 neural stem cells are also induced into neuronal cells by miR-124a, the different concentration of miR-124a (10, 30, and 50 nM) was treated to F3 cells. Consistent with F11 cells, Tuj1 expression increased in miR-124a-treated F3 cells in the cytoplasmic region from 10 nM concentration, compared to miR-scramble-treated cells (Fig. 3).

### Time course of F11 cell changes showing neuronal-lineage differentiation by miR-124a on fluorescence live cell imaging in vitro

**Fig. 4:**
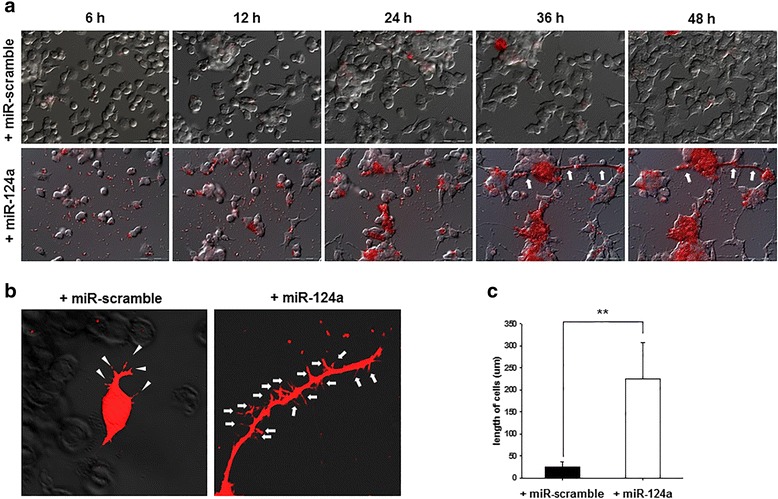
Neuronal differentiation of F11 cell changes by miR-124a on fluorescence live cell imaging. MiR-124a or miR-scramble was co-transfected with pTα1-RFP into F11 cells, and the changes of RFP reporter expression were examined every 30 min during 48 h. **a** RFP signal increased gradually in F11 cells treated with miR-124a, but not in miR-scramble treated cells. **b**, **c** The length of neurite outgrowth in miR-124a-transfected F11 cells was longer than miR-scramble-transfected cells

Once we introduced alpha tubulin 1 (pTα1-RFP) reporter to F11 cells [39] and observed once every 30 min until 48 h after, RFP signals increased gradually in miR-124a-treated F11 cells. At 36 h, RFP signals were prominent along with neurite elongation in the miR-124a-treated F11 cells (Fig. 4a). The length of neurite outgrowth was measured by a Zeiss 510 Meta Laser scanning microscope, and the outgrowth length was much longer in miR-124a-treated F11 cells than miR-scramble-treated F11 cells (Fig. 4b, c).

### In vivo fluorescence imaging of differentiating F11 cells induced by co-administration of miR-124a to the implants

**Fig. 5:**
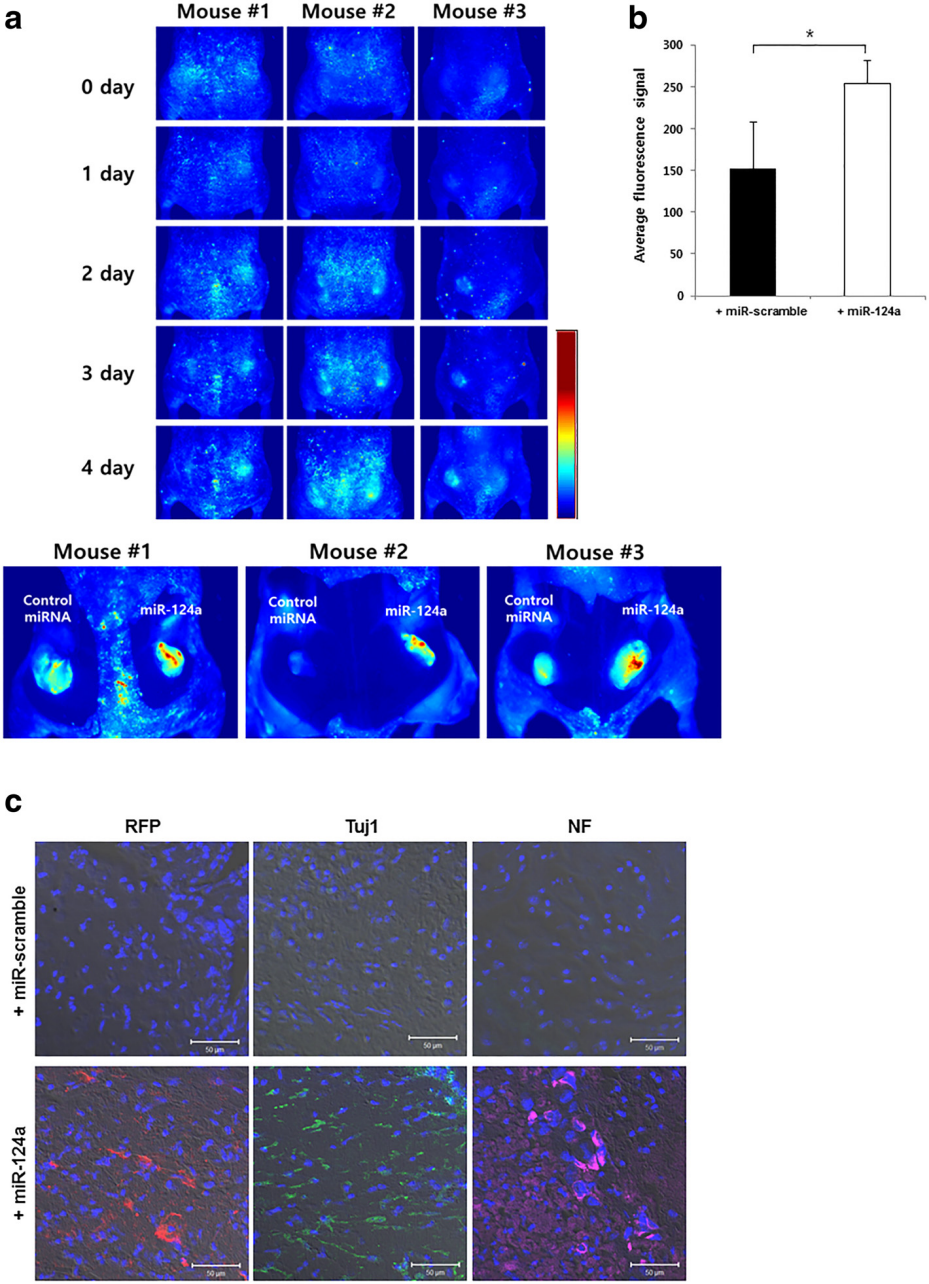
In vivo fluorescence imaging in the differentiated F11 cells induced by miR-124a. F11 cells transfected with pTα1-RFP were subcutaneously inoculated into both thighs of nude mice with matrigel (*n* = 3). Before injection, F11 cells were mixed with miRNAs (miR-124a or miR-scramble) coated with in vivo-jetPEI™ solution. **a** Fluorescence images were daily acquired in individual group up to 4 days. RFP signals in miR-124a-treated F11 cells were higher in nude mice at 4 days, compared to those in miR-scramble-treated F11 cells. **b** After the mouse skin was removed at 4 days, ROI analysis demonstrated higher fluorescence signals in miR-124a-treated cells than miR-scramble-treated cells (*n* = 3 *p < 0.05). ROI area was drawn on each injection site. **c** F11 cells were isolated from the injection sites from the sacrificed nude mice, and immunohistochemistry was done for RFP and neuronal markers (Tuj1 and NF). MiR-124a-treated F11 cells showed increased RFP, Tuj1, and NF levels, compared with miR-scramble-treated group

After pTα1-RFP-transfected cells were mixed with in vivo-tailored PEI-coated miR-124a or miR-scramble, the transfected cells were subcutaneously injected in the left and right thighs of mice, and in vivo fluorescence images were acquired daily for 4 days in nude mice. Background fluorescence was high as soon as the cells were implanted (0 day). However, fluorescence signals increased at 2 and 4 days in the right side where miR-124a was co-administered (Fig. 5a). After removing the skin at 4 days, fluorescence of the miR-124a-co-administered side was distinctly higher than that of the miR-scramble-co-administered side. The semi-quantitative region of interest (ROI) value revealed the higher fluorescence signals in miR-124a-treated F11 cell group at 4 days (Fig. 5b). To verify the neuronal differentiation pattern of F11 cells by overproduction of miR-124a in vivo, we isolated F11 cells from injected sites of the mice immediately after sacrifice the mice.

Immunohistochemistry analysis showed that expression of Tuj1, early neuronal marker and NF, and late neuronal markers increased in miR-124a-treated F11 cells, compared to those in miR-scramble-treated F11 cells (Fig. 5c).

## Discussion

Facilitating the neuronal differentiation from the grafted stem or progenitor cells is important to enhance therapeutic efficacy of the stem cell treatment for a variety of neurological diseases. There are many types of already known molecules enhancing neuronal differentiation such as a chemical compound (retinoic acid, cAMP), exogenous genes (neuroD2, ngn1), and non-coding RNAs [12, 13, 40–42]. Several miRNAs have been reported closely linked to regulating neuronal differentiation in the development of neurological diseases [43–45]. For example, the down-regulation of neurogenic miRNAs such as miR-9 and miR-132 leads to the impaired neurogenesis [46, 47]. MiR-124a is well-known as an essential regulator of neuronal differentiation by down-regulating the proliferation-related genes [48]. Using miR-124a to promote neuronal differentiation may be a feasible strategy considering its definite expression difference in neural progenitor cells between pre-and post-neuronal differentiation [49, 50]. In addition, several reports demonstrated that miR-124a itself was sufficient to induce neuronal differentiation, which can even be regarded as an accelerator to induce neurogenesis [51, 52]. We assumed that the up-regulation of miR-124a expression increase the neuronal differentiation helping enhance therapeutic efficacy of stem/progenitor cell implants. If we want to prove this strategy works or not, it is important to reveal how much of the implanted progenitor cells (or stem cells) differentiate into neuronal lineage by miR-124a administration and molecular imaging method we used worked and was simple and reliable.

In our results, the immunofluorescence clearly revealed that miR-124a facilitated the differentiation toward neuronal lineage in a short period of time in neural progenitor F11 or neural stem F3 cells. The early neuronal marker Tuj1 and late neuronal marker NF levels were definitely increased in miR-124a-treated F11 cells, showing elaborate neurite formation (Fig. 2b). We also examined the dynamic time courses of reporter expression representing neuronal differentiation of F11 cells induced by miR-124a and live F11 cells provided in vitro their extent of differentiation efficiency. Neuron-like morphological changes of F11 neuronal progenitor cells after the overexpression of miR-124a complied with the Tα1 promoter-driven RFP reporter gene expression visualizing the neuronal differentiation of these cells [53]. MiR-124a-transfected F11 cells clearly showed the gradual increase in RFP signals along with stretched cell body in live cell condition until 2 days after miR-124a transfection.

For in vivo miR-124a transfection study, we used easily available in vivo jetPEI™, which is composed of a positive charged polyethylenimine polymer, and mixed our miR-124a with this polymer transfection agent [54]. When RFP-laden F11 cells were co-administered with PEI-miR-124a complex as an implant into each thigh of nude mouse, the similarly low RFP signals and excessive auto-fluorescence signals from the skin were observed both in miR-124a-treated and miR-scramble-treated side at 0 day. However, the fluorescence intensity in miR-124a-treated F11 cell group became higher at 4 days, compared to scramble-treated F11 cell group. In in vivo fluorescence imaging study in skin-removed mice, visually and quantitatively, the discrepancy of RFP signal between miR-124a-treated and scramble-treated group became prominent at 4 days. *Ex vivo* study has also confirmed that the implanted F11 cells differentiated to the neuronal lineage by the influence of miR-124a co-administered mixed with PEI in vivo. Immunohistochemistry proved the miR-124a-induced increased expression of Tuj1 and NF in the isolated section of implanted F11 cells. These finding demonstrated that exogenous miR-124a oligomer alone, when co-administered in matrix gel with the cell implants, induces the neuronal differentiation of F11 cells in nude mice. This phenomenon could be examined simply using in vivo fluorescence imaging technique.

The imaging investigation of the functional outcome of miRNA co-administration will be helpful for the evaluation of the possible in vivo effect of various miRNA or related nucleic acid oligomers (such as siRNA or gapmer). Some of them might be as a key controller of a variety of biological phenomenon such as cell differentiation. Though we just proved the effect of single microRNA (miR-124a) in this study, PEI system easily hosts cocktails of microRNAs. This easy sensing technique to report miRNA function in vivo will be attractive because of widespread applicability for elucidating unknown functions of specific miRNA.

Fluorescence-based optical imaging modality has been widely harnessed to examine time-course changes of amount of biomolecules with high resolution. Nevertheless, the intrinsic shortcoming that cannot acquire the signals in deep tissue hampers to move forward clinical application. Much effort has been made to use a longer NIR wavelength and wavefront shaping technique with the control of multiple light scattering to overcome tissue penetration issue [55, 56]. Even though our fluorescence-based approach to trace the effect of miR-124a might not give useful information non-invasively in clinic, this technique could be applied for minimal invasive intraoperative study using multiphoton microscopy to trace the course of neuronal differentiation by miR-124a in living brain tissue.

## Conclusions

In this study, we imaged the functional outcome of miR-124a transfection to the stem/progenitor cell implants focusing on the activating the neuronal differentiation using neuron-specific promoter-based reporter gene system. In vitro live cell tracking to trace the dynamic changes of differentiation toward neuronal fate by miR-124a would have also inform us when we can expect the probable changes of cellular fate after in vivo administration despite the difference between in vitro and in vivo situation. We suggest that this approach provides invaluable information to evaluate functional outcome of cell-based therapy co-administered with neurogenic miRNAs in a variety of neurological disease model.
